# Molecular interaction of silicon quantum dot micelles with plasma proteins: hemoglobin and thrombin †

**DOI:** 10.1039/c9ra02829c

**Published:** 2019-05-14

**Authors:** Shanmugavel Chinnathambi, Subramani Karthikeyan, Nobutaka Hanagata, Naoto Shirahata

**Affiliations:** International Center for Young Scientists, National Institute for Materials Science (NIMS) 1-2-1 Sengen Tsukuba Ibaraki 305-0047 Japan CHINNATHAMBI.Shanmugavel@nims.go.jp; Department of Organic Chemistry, Peoples' Friendship University of Russia (RUDN University) MikluhoMaklaya St., 6 Moscow 117198 Russia; International Center for Materials Nanoarchitectonics (WPI-MANA), NIMS 1-1 Namiki Tsukuba 305-0044 Japan; Nanotechnology Innovation Station, NIMS 1-2-1 Sengen Tsukuba Ibaraki 305-0047 Japan SHIRAHATA.Naoto@nims.go.jp

## Abstract

Protein conformational changes are associated with potential cytotoxicity upon interaction with small molecules or nanomaterials. Protein misfolding leads to protein-mediated diseases; thus, it is important to study the conformational changes in proteins using nanoparticles as drug carriers. In this study, the conformational changes in hemoglobin and thrombin were observed using fluorescence spectroscopy, circular dichroism spectroscopy and molecular modelling studies after interaction with non-toxic, water-soluble near-infrared silicon quantum dot micelles. The molecular docking results indicated that the binding affinities of hemoglobin and thrombin with Si QD micelles are good. In addition, molecular dynamics simulations were performed to obtain more detailed information.

## Abstract 2

Overall graphical representation of 1-decene, F-127, and crystal structures of hemoglobin and thrombin.

## Graphical abstract

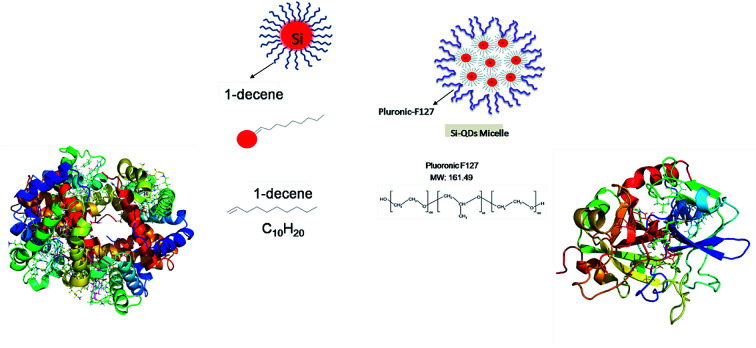

## Introduction

Quantum dots (QDs) are excellent fluorescent materials for bio-imaging, tracking and drug delivery applications, but very few studies are available for their interactions with biomolecules.^1–5^ Compared with commercially available organic dyes, semiconductor QDs are highly stable against photobleaching and have special properties such as a high quantum yield, broad absorption, and a narrow and tunable emission.^6^ The toxicity of QDs is a critical factor because of the release of heavy metal ions after delivery into the bloodstream.^7^ When QDs are injected into the blood vessels intravenously, they are rapidly surrounded (adsorption) by biomolecules, particularly plasma proteins. The surface of the nanomaterials becomes covered by the proteins, and this is called a protein corona.^8^ In contrast, the adsorption of proteins onto the surface of QDs alters the secondary/tertiary structures of the proteins, leading to toxicity. Many reports indicate that silicon QDs are nontoxic for bio-environments.^9–13^ Here, we prepared water-soluble and nontoxic near-infrared Si QD micelles to clarify the interaction mechanism with hemoglobin and thrombin.

Blood plasma proteins are the main target of several medicines, metal ions, viruses, *etc.* Hemoglobin is a tetrameric protein; each monomer contains a heme group, which is responsible for binding and releasing O_2_ and CO_2_ to the cells and lungs. Two identical alpha chain (141 amino acids) and two identical beta chain (146 amino acids) subunits are stabilized by hydrogen bonds.^14^ Thrombin is a serine protease containing two positively charged terminal domains called exosites 1 and 2. In the terminal, the exosites can bind many cofactors and substrates such as fibrinogen and thrombomodulin in exosite 1 and heparin and the platelet receptor GPlba in exosite 2.^15^ Recently, the interaction of nanoparticles with proteins and protein corona formation on nanomaterials have been actively researched.^16–18^ The mechanism of nanomaterials binding with proteins is a critical factor to understand the functions of nanomaterials in the human body. The affinity between the nanoparticles and proteins plays a critical role. A lower binding affinity shows poor distribution and short life-span, whereas higher affinity indicates cytotoxicity and conformational changes. Hence, the aim of this article was to explore the binding mechanism of the Si QD micelles on hemoglobin and thrombin by means of biophysical and molecular dynamic approaches.

## Materials and methods

### Materials

Hemoglobin (H7379) and thrombin (T4393) were purchased from Sigma-Aldrich. The purity of the proteins was verified by a UV-vis nanodrop spectrophotometer. Triethoxysilane (TES), 1-decene, and mesitylene were purchased from Sigma Aldrich. All other chemicals were purchased from Wako Pure Chemical Industries Ltd (Japan) and used as received. Water was purified and deionized using a Sartorius (arium 611 UV) water purification system. NIH3T3 fibroblast cell lines (ATCC® CRL-1658) were obtained from ATCC.

### Synthesis of Si QD micelles

Initially, 16 mL of TES was placed in a round bottom flask, which was then placed in an ice bath; the solution was stirred under an argon atmosphere. Then, 32 mL of acidic water (HCl – pH 3) was added dropwise to TES with stirring. After xerosol formation, it was dried under vacuum. The white solid was placed into a quartz crucible, transferred to a high-temperature furnace, and heated at 1150 °C for 2 h under 10^−4^ Pa pressure and an inert atmosphere. The black powder was ground into a fine powder (HSiO_1.5_)_*n*_ using a mortar and a pestle. To get free-standing silicon quantum dots, the fine (HSiO_1.5_)_*n*_ powder (300 mg) was placed into a small Teflon container and slurried in a mixture of ethanol (10 mL), HF (48%, 10 mL) and water (10 mL).^19^ The mixture was stirred for about 80 minutes to remove the SiO_*x*_ matrix using the acidic etching method. Hydrogen-terminated Si QDs were recovered by centrifugation at 1500 rpm for 5 minutes and washed with ethanol two times. The washed Si QDs were moved into a Schlenk flask containing mesitylene (15 mL) and 1-decene (15 mL). The mixture was degassed 3 to 5 times with Ar, heated at 170 °C for 2 hours, and then cooled to room temperature. The resulting 1-decene-coated Si QDs were mixed with toluene/methanol and centrifuged for about 40 minutes at 15 000 rpm to remove excess mesitylene and unreacted 1-decene. The product dried under vacuum was then dispersed in 3 mL of chloroform. 1-Decene-functionalized Si QDs (3 mL) in chloroform were purified using high-performance liquid chromatography (HPLC) with a flow rate of 3.5 mL min^−1^. Thirty minutes later, we collected the 1-decene-functionalized Si QD samples and then, chloroform was removed using a rotary evaporator. The purified 1-decene-functionalized Si QD samples were diluted with 5 mL toluene for further use.

### Characterizations

UV-visible absorption spectra were recorded on a JASCO V-7200 UV-vis spectrophotometer. HR-TEM measurements were obtained from a 300 kV field emission transmission electron microscope (Tecnai G2 F30). Samples for HR-TEM analyses were drop-casted from dilute dispersions of Si QDs in ultrathin-carbon (<10 nm thickness)-coated copper grids. PL and PLE spectra were obtained with a NanoLog Horiba Jobin Yvon spectrofluorometer using an InGaAs detector for NIR (Hamamatsu Photonics Co. Ltd, Japan) at room temperature. Time-resolved fluorescence decay profiles were recorded at room temperature on a time-correlated single photon counting (TCSPC) lifetime system (NanoLog Horiba Jobin Yvon, Japan) equipped with pulsed laser diodes (*λ*_em_ = 370 nm). The quality of the fit was assessed based on the *χ*^2^ value (∼1.0) and a visual inspection of the residuals. Absolute PL quantum yields were measured using a C9920-03G system equipped with a 150 W xenon lamp (Hamamatsu Photonics Co. Ltd, Japan). Dilute solutions having absorptions between 0.1 and 0.2 were inserted into the instrument sample compartment with 1 cm^2^ quartz cuvettes. UV-visible absorption spectra were recorded with a Jasco V-650 spectrophotometer. The CD spectra of all plasma proteins and those of proteins complexed with micelles were recorded with a spectropolarimeter (model J-725; Jasco, Tokyo, Japan) using a 1 mm quartz cuvette with a scan speed of 100 nm min^−1^ at 25 °C. Each spectrum shows an average of three scans. All the protein secondary structure predictions were calculated using a web server BeStSel.

### Cell cultures

NIH3T3 cells were cultured in a 75 cm^2^ flask for the cytotoxicity assay and fluorescence microscopy observation. The NIH3T3 cells were maintained in a Dulbecco's Modified Eagle Medium with low glucose supplemented with fetal bovine serum (10%), penicillin (50 U mL^−1^) and streptomycin (50 mg mL^−1^) at 37 °C in humidified air containing 5% CO_2_.

### Cellular uptake and confocal microscopy

To analyze the intracellular localization of the Si QD micelles, we added them at a final concentration of 100 μg mL^−1^ to NIH3T3 cells cultured in a 35 mm dish for 24 h. The cells were then washed 3 times with PBS and fixed with 3.7% formaldehyde for 20 min. The differential interference contrast and fluorescence images were obtained with a confocal laser scanning fluorescence microscope (SP5, Leica Microsystems, Germany) under a UV-LED laser. Also, we observed the Si QD micelles directly under a confocal microscope using the same laser source.

### Molecular docking

The initial chemical structure of 1-decene with Si and F-127 was drawn using the ChemDraw software. The primary structure of the chemical components was energy minimized using the steepest descent and followed up with a conjugate gradient algorithm for 5000 cycles, which is inbuilt in the impact panel in the Schrödinger software. The crystal structures of hemoglobin (PDB ID: 1ppb) and thrombin (PDB ID: 1gzx) were downloaded from the protein data bank portal (http://www.rcsb.org/pdb). Both the obtained protein structures were optimized and minimized using the optimized potentials for a liquid simulations (OPLS 2005) force field, which is in the protein preparation wizard panel. The protein preparation method was done using the following steps: (i) all water molecules around the protein molecules were removed; (ii) hydrogen atoms were added into the respective protein structure; (iii) Coulomb charges were also assigned; (iv) finally, the native (co-crystal) molecules were removed from the respective protein molecules and the structure was energy minimized. Finally, the docking studies were carried out using the induced fit panel and maximum 20 different poses were generated; the best docking pose was selected based on the glide energy and docking score.^20–26^

### Molecular dynamics

The best docked poses of the 1-decene complex with hemoglobin and thrombin and the F-127 complex with hemoglobin and thrombin were taken for further molecular dynamic studies. The primary protein–ligand complex file was prepared using the Desmond system builder panel, which is inbuilt in the Schrödinger suite. Here, all the protein–ligand complex systems were prepared with 10 Å^3^ cubic boxes from the center of the mass with the super molecule. The TIP3P water solvation system was used as the buffer system with the charged ions placed isotopically to neutralize the Ewald charge summation of the solvated protein entity. The system was minimized with the maximum number of iterations of 5000 steps with a gradient convergence threshold of 1.0 kcal mol^−1^ Å^−1^. Once the system was minimized, it was subjected to Newtonian dynamics of the model system to evaluate the energy of the system. Also, 2 ps steps were integrated to record the simulation. A six-stage NPT ensemble default relaxation process was carried out before performing the molecular dynamics simulation. The initial state solute-restrained Brownian dynamics of the ensemble were carried out by keeping the energy constant using the NVT condition. In the second stage, using the Berendsen thermostat, the NVT (canonical) ensemble was allowed to relax with respect to the temperature with the velocity resembling of every 1 ps applied to the non-hydrogen solute sample. Subsequently, the NVT ensemble was changed to an NPT ensemble with the Berendsen Barostat and the system was kept at 1 atm pressure, followed by system equilibration of 1 ns. Then, the ensemble was subjected to a 20 ns molecular dynamics run.^27,28^

## Results and discussion

### Biophysical analysis

**Fig. 1:**
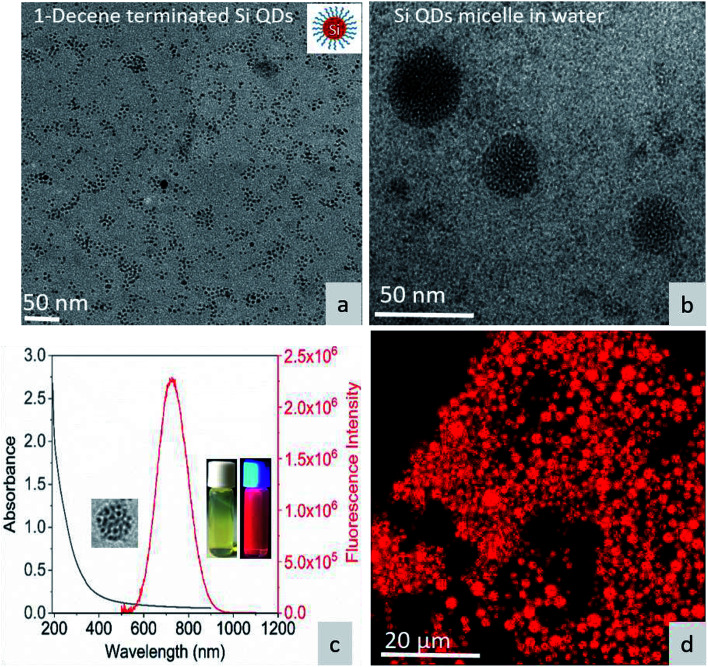
(a) TEM image of 1-decene-terminated Si QDs. (b) TEM image of F-127/Si QD micelles in water. (c) UV-visible absorption and emission spectra of the micelle – inset show micelle under sample under white light and UV light. (d) Micelle structure under confocal fluorescence microscopy.

Fig. 1a shows the TEM image of surface-functionalized Si QD distribution in toluene. The TEM image in Fig. 1b shows the Pluronic F-127-coated Si QD micelles having a size less than 50 nm. The photoluminescence of the micelles showed a sharp emission peak around 675 nm with a broad absorption spectrum in the UV region. The inset in Fig. 1c shows a red color emission coming from the Si QD micelles upon excitation with a 365 nm UV light. In addition, the red emission was confirmed by confocal microscopy during excitation with the UV laser (Fig. 1d). Fig. S1† shows that the hemoglobin and thrombin absorption was enhanced on increasing the concentration of Si QD micelles. The increment in absorption showed that there is binding between the micelle surface and the amino acids present in the proteins. In particular, tryptophan residues exhibit absorption around 280 nm and the π–π* transition of the polypeptide backbone of C=O shows a highly intense peak around 200 nm.^29^ This observation suggests that ground state complex formation occurs during the interaction between the micelles and proteins.^30^

**Fig. 2:**
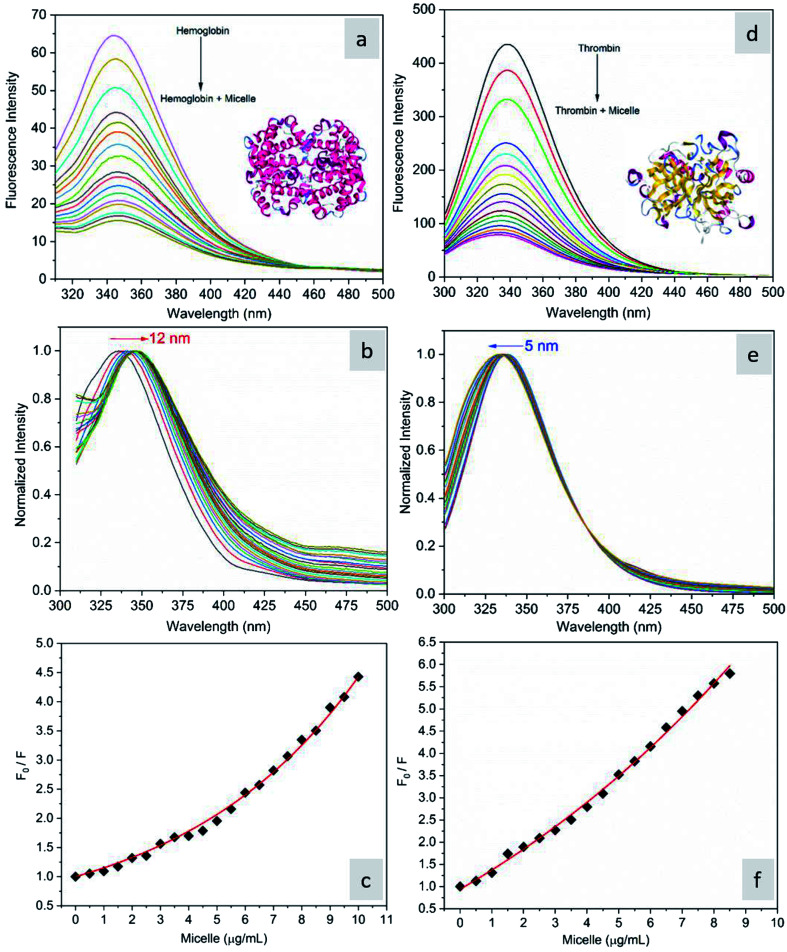
(a) Fluorescence emission spectra of hemoglobin (2 μM) alone and hemoglobin with increasing concentrations of micelles, (b) its normalized spectrum, and (c) the corresponding Stern–Volmer plot. (d) Fluorescence emission spectra of thrombin (2 μM) alone and thrombin with increasing concentrations of micelles, (e) its normalized spectrum and (f) the corresponding Stern–Volmer plot.

**Fig. 3:**
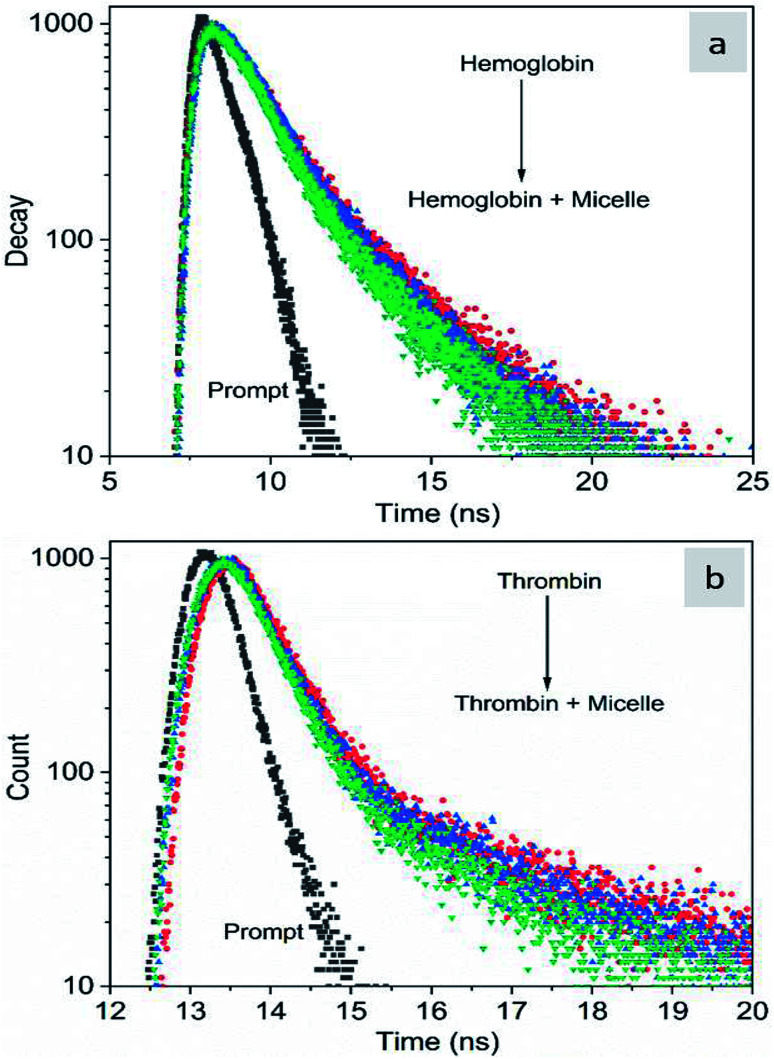
Time-resolved fluorescence decay profiles of hemoglobin (a) and thrombin (b) with increasing concentrations of Si QD micelles.

The fluorescence quenching of molecules is due to several processes such as energy transfer, ground state complex formation and collisional quenching. They are classified into two processes: static quenching and dynamic quenching. In order to understand the binding mechanism of the proteins with the micelles and to know the nature of quenching, the fluorescence spectra of the proteins in conjugation with different concentrations of micelles were recorded at 280 nm excitation (Fig. 2). From Fig. 2a and d, it is observed that the fluorescence intensity decreases upon addition of micelles in addition to a spectral shift. In the case of hemoglobin, after 10 μg mL^−1^ of micelle addition, the emission peak shifted towards a longer wavelength (12 nm), while thrombin showed 5 nm blue shift (Fig. 2b and e). The fluorescence quenching and spectral shift confirmed that there is binding between proteins and micelles and hydrophobic and hydrophilic environmental changes occur during the interactions. Fig. 2c and f show a linear trend at lower concentrations and a non-linear trend at higher concentrations of micelles. Static quenching is present at lower concentrations and dynamic collisional quenching is present at higher concentrations of the micelles. The fluorescence lifetime measurements can give information about the conformational heterogeneity of the proteins and can help distinguish between the static and dynamic processes.^31^ We analyzed the dynamic nature of both proteins during interactions; the average lifetime of hemoglobin varied from 1.74 ns to 1.41 ns and that of thrombin from 3.18 ns to 1.44 ns (Fig. 3a and b). The lifetime variation of the components and their average showed the changes in the microenvironment (pH) of the protein and the protein–micelle complex.

**Fig. 4:**
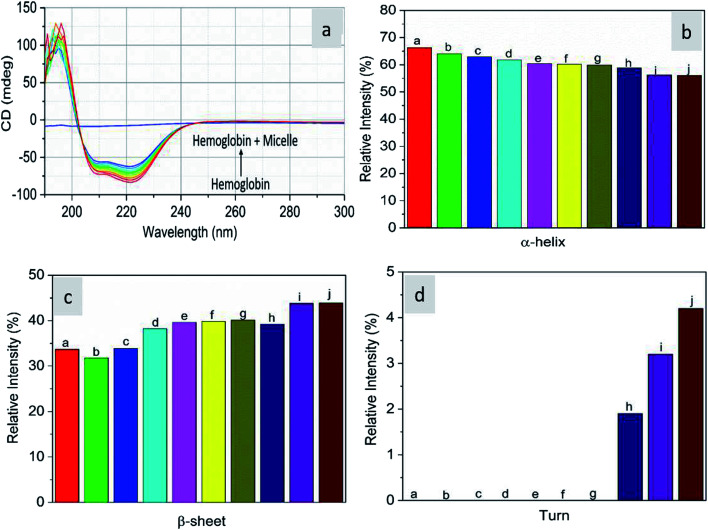
Far-UV CD spectra of hemoglobin in the absence and presence of different concentrations of micelles.

**Fig. 5:**
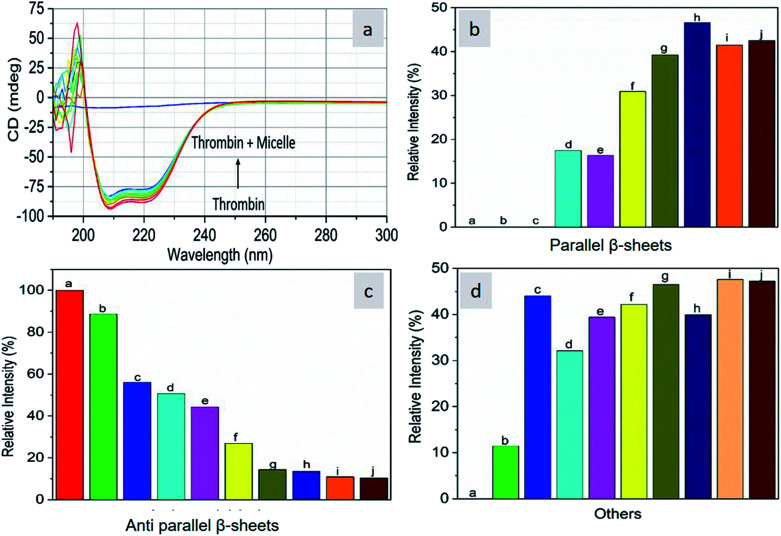
Far-UV CD spectra of thrombin in the absence and presence of different concentrations of micelles.

As CD is one of the most sensitive methods for monitoring the conformational changes in proteins, the CD spectra of the proteins with and without micelles were recorded. The data of all the secondary structures of proteins were analyzed using the BESTSEL online software (http://bestsel.elte.hu/index.php).^32,33^Fig. 4 displays the CD spectra of the proteins and protein complexes with the micelles. From the figure, two negative bands at 208 and 222 nm are observed. These bands are attributed to the n–π* transition of the peptide bond of the α-helical structure of the protein. From these results, it is found that the percentage of α-helicity decreases with the increase in micelle concentration. For example, the percentage of α-helicity for pure hemoglobin (Fig. 4a–d) is found to be 66.30%. In the presence of increasing concentrations of micelles, the α-helix percentages were found to be 64%, 62.9%, 61.8%, 60.4%, 60.2%, 59.9%, 58.9%, 56.2%, and 56.1%. At the same time, the thrombin (Fig. 5a–d) beta sheet reduced from 100 to 52%, indicating that the binding of the micelles with the amino acid residues decreases with the α-helical and beta sheet contents. However, the shape and the peak position in the CD spectra indicate that the original structure of the protein does not change even after binding with micelles. From the literature, it is evident that this type of interaction may not alter the secondary structure of proteins (α-helix and β-sheet), which is the prime requirement for biomedical applications.

### Molecular docking analysis

**Fig. 6:**
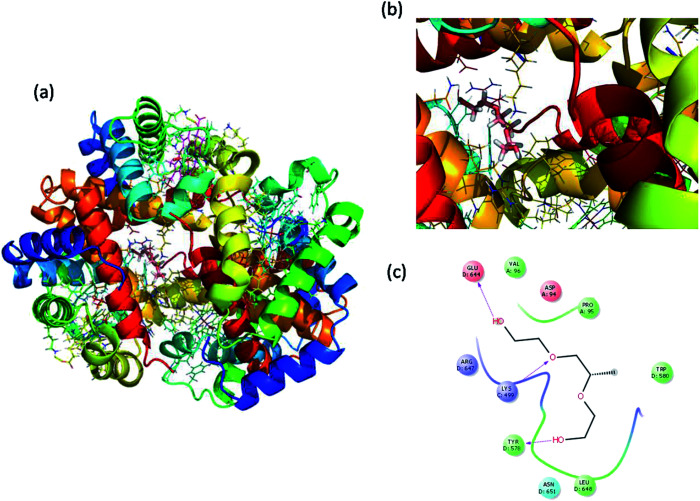
(a) Best binding pose of hemoglobin with the F-127 complex. (b) Interior of the F-127 complex in hemoglobin. (c) The interaction plot of the F-127 complex in hemoglobin.

**Fig. 7:**
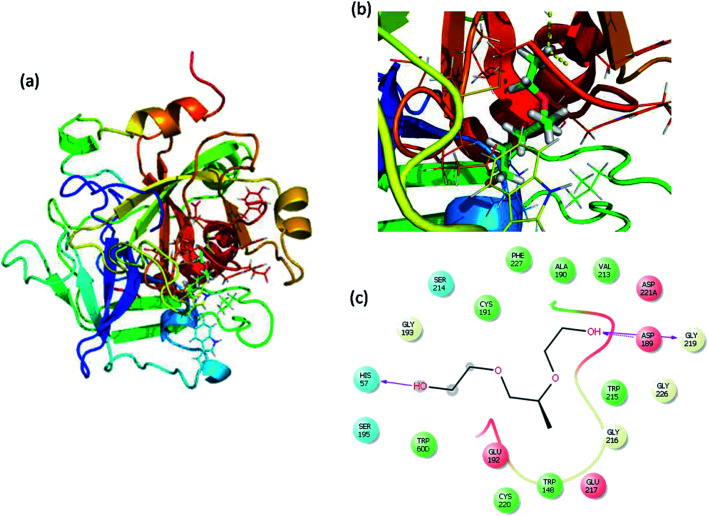
(a) Best binding pose of thrombin with the F-127 complex. (b) Interior of the F-127 complex in thrombin. (c) The interaction plot of the F-127 complex in thrombin.

Molecular docking is a great tool for understanding complex biological systems at an atomic level. After the experimental binding confirmation of 1-decene and F-127 in hemoglobin and thrombin systems, molecular docking studies were carried out to obtain more insights into the binding interaction mechanisms of 1-decene and F-127 complexes with hemoglobin and thrombin microenvironments. Blind docking was performed to find the active site residues. Based on the glide energy, the best binding pose was determined. From this complex, the active site residues were taken to conduct further induced-fit docking between 1-decene and F-127 complexes with hemoglobin and thrombin systems. After the induced fit docking simulation, 20 different binding poses were generated. Based on the glide energy and docking score, the best poses were taken for further analysis of the 1-decene and F-127 complexes with hemoglobin and thrombin systems. Fig. S2, S3,†6 and 7 show the best binding positions of the 1-decene complex with hemoglobin and thrombin and those of the F-127 complex with hemoglobin and thrombin systems. The results show that 1-decene complexes with both hemoglobin and thrombin are mainly due to the hydrophobic interactions, and the binding energies are −30.5 kcal mol^−1^ and −26.8 kcal mol^−1^, respectively. In the case of the F-127 complexes with the hemoglobin and thrombin protein systems, the binding energies are −29.7 kcal mol^−1^ and −26.9 kcal mol^−1^, respectively, and three hydrogen bonds are formed between the D and C chains of the hemoglobin–F-127 complex (Fig. 6c) (D: Glu 644, D: Tyr 578 and C: Lys499). In the thrombin–F-127 complex system (Fig. 7c), it is also shown that there are three hydrogen bonds formed among the His 57, Asp 189, and Gly 219 residues. The overall docking result indicates that 1-decene and F-127 bind very well to the hemoglobin and thrombin protein molecule systems.

### Molecular dynamic analysis

Molecular dynamics simulations were carried out to understand the stability of 1-decene and F-127 in hemoglobin and thrombin protein molecule systems. The Molecular Dynamics (MD) simulations generate Root Mean Square Deviation (RMSD), Root Mean Square Fluctuation (RMSF) and Secondary Structural Element (SSE) reports for the 1-decene and F-127 complexes with hemoglobin and thrombin systems. Fig. S4a and S4b† show RMSD of 1-decene with the hemoglobin complex system; Fig. S4c and S4d† show RMSD of the F-127 complex in thrombin. In this figure, the protein RMSD (Cα) is first aligned with the reference backbone and RMSD is further calculated based on atom selection. Monitoring the protein RMSD results will give more structural confirmation throughout the simulation. From the results in Fig. S4,† it is shown that the 1-decene and F-127 (Fig. S4a and S4c†) complexes with hemoglobin have thermal average structural fluctuation changes between 1 and 3 Å; this result is acceptable for small, globular proteins. However, in the case of the 1-decene and F-127–thrombin complexes (Fig. S4b and S4d†), the RMSD results show a larger structural fluctuation and indicate that both protein complexes undergo larger conformational changes during simulation. Ligand RMSD (Lig fit Prot) indicates the stability with the respective protein complexes during simulation. Fig. S5a and S5b† show RMSF of 1-decene with hemoglobin complex system, and Fig. S5c and S5d† show RMSF of the F-127 complex in thrombin. The RMSF result is useful for characterizing local changes along the protein chain. In this figure, 12 ligand contacts with the protein residues that interact with the ligand are marked with green-colored vertical bars and the RMSF B-factor of the protein can also be correlated with the experimental X-ray B-factor. With this result, we conclude that in Fig. S5,† the peaks indicate the areas of the protein that fluctuate the most during simulation. Protein secondary structure elements (SSE) such as alpha-helices and beta-strands were monitored throughout the simulation for 1-decene and F-127 complexes with hemoglobin and thrombin, as shown in Fig. S7† as well as in Fig. S8–S11.† These interactions can be categorized by type and summarized, as shown in the plot above. The protein–ligand interactions are categorized into four types: hydrogen bonds and hydrophobic, ionic and water bridges (Fig. S6† and 8). The stacked bar charts are normalized over the course of the trajectory: for example, a value of 0.7 suggests that the specific interaction is maintained for 70% of the simulation time. Values over 1.0 are possible as some protein residues may make multiple contacts of the same subtype with the ligand. Each interaction type contains more specific subtypes, which can be explored through the simulation interaction diagram (Fig. S13–S16†). In addition, the ligand RMSF may give insights on how the ligand fragments interact with the protein and their entropic role in the binding event (Fig S12†). Finally, the ligand torsion plot summarizes (Fig. S17–S20†) the conformational evolution of every rotatable bond (RB) in the ligand throughout the simulation trajectory. The molecular dynamics simulation event concludes that the binding of 1-decene and F-127 in hemoglobin and thrombin protein molecule systems is good and this result may support the above experimental suggestions.

**Fig. 8:**
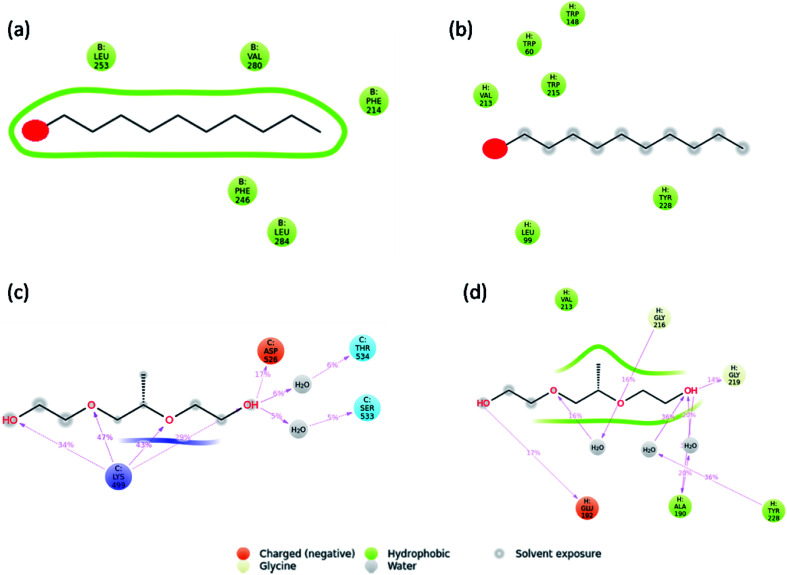
Interaction plots after simulations of the (a) hemoglobin complex with 1-decene, (b) thrombin complex with 1-decene, (c) hemoglobin complex with F-127, and (d) thrombin complex with F-127.

## Conclusion

In summary, detailed NIR-emissive Si QD micelle behaviors in both hemoglobin and thrombin were discussed using various spectroscopies, molecular docking and molecular dynamic studies. The steady-state fluorescence spectroscopy analysis showed that the micelles quench both the proteins by static quenching in lower concentrations and by dynamic quenching at higher concentrations. The CD analysis showed that the secondary structural changes of the plasma proteins are not perturbed upon micelle binding over the concentration range. Molecular docking studies indicated that F-127 binds very well to the hemoglobin and thrombin protein molecule systems. The molecular dynamics simulation showed that the binding of F-127 to the hemoglobin and thrombin protein molecule systems is good, and this result will support the experimental results. These experimental and simulation results will be helpful for developing nontoxic NIR biomarkers for *in vivo* trials in the near future.

## Conflicts of interest

There are no conflicts to declare.

## Supplementary Material

RA-009-C9RA02829C-s001
