# Supplementary material for: Molecular interaction of silicon quantum dot micelles with plasma proteins: hemoglobin and thrombin
Source: RSC Adv. 2019 May 14;9(26):14928–36. doi: 10.1039/c9ra02829c (PMC9064248; doi:10.1039/c9ra02829c)
Supplement: RA-009-C9RA02829C-s001 [file RA-009-C9RA02829C-s001.pdf]

Electronic Supplementary Material (ESI) for RSC Advances.
This journal is © The Royal Society of Chemistry 2019

Supporting Information

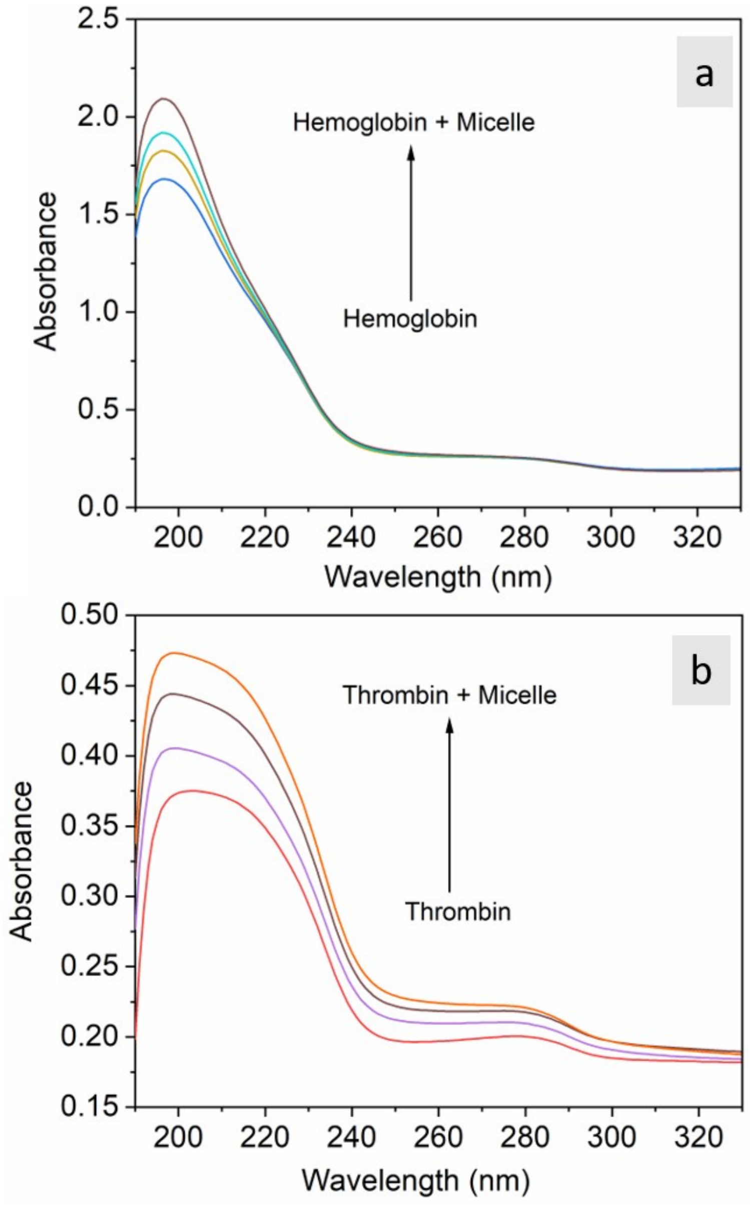

Figure.S1 a). UV-Visible absorption spectra of hemoglobin (2 μM) alone and hemoglobin with increasing concentration of the micelle. b). UV-Visible absorption spectra of thrombin (2 μM) alone and thrombin with increasing concentration of the micelle.

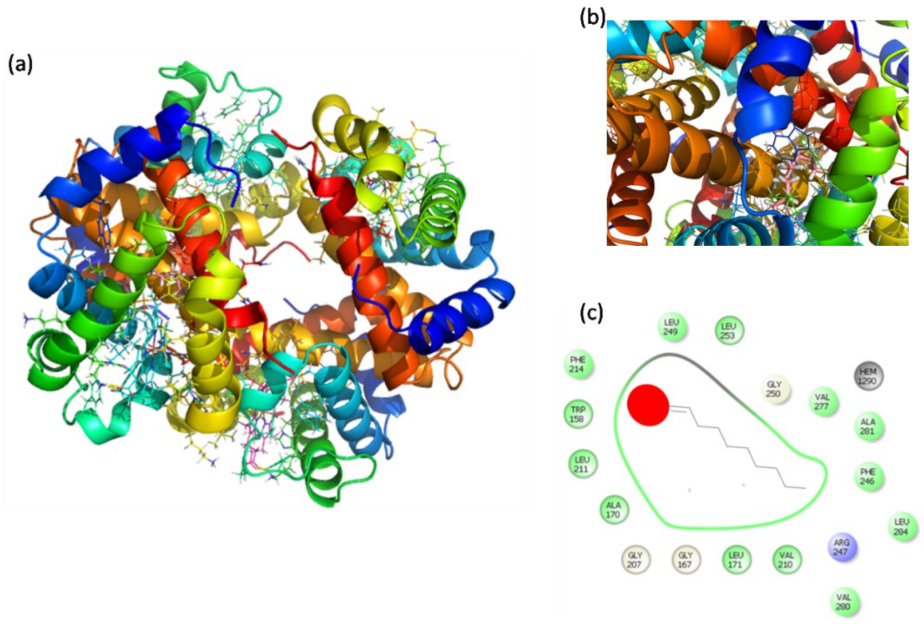

Figure.S2 (a) Best binding pose of hemoglobin with 1 decene complex (b) inset of 1 decene complex in hemoglobin (c) show the interaction plot of 1 decene complex in hemoglobin.

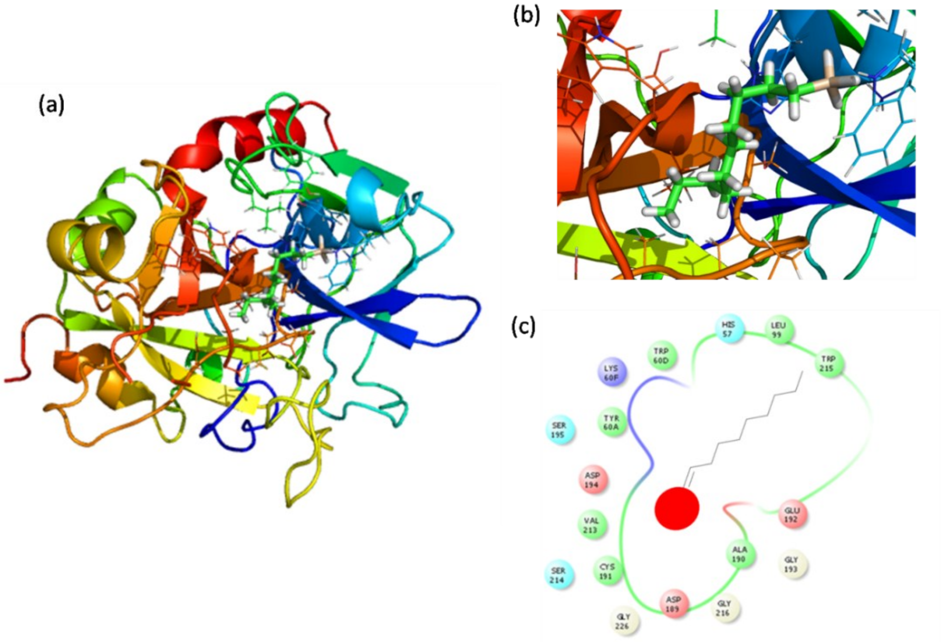

Figure.S3 (a) Best binding pose of thrombin with 1 decene complex (b) inset of 1 decene complex in thrombin (c) show the interaction plot of 1 decene complex in thrombin.

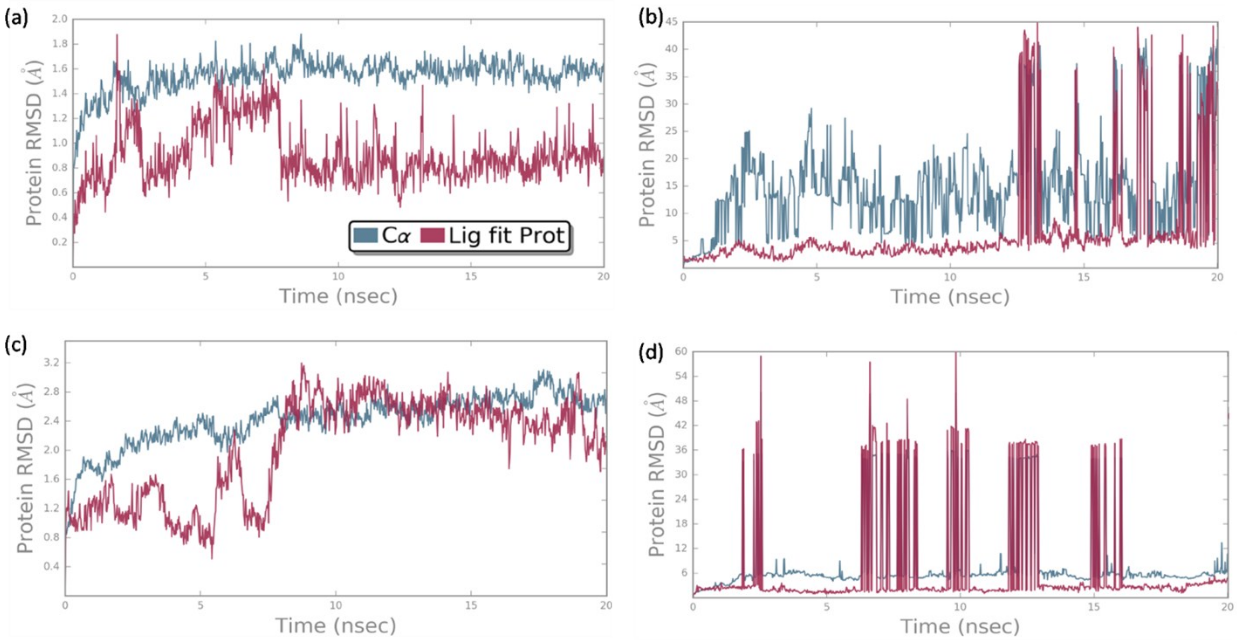

Figure. S4 Root mean square deviation plot for (a) hemoglobin complex with 1 decene (b) thrombin complex with 1 decene (c) hemoglobin complex with f127 (d) thrombin complex with f127.

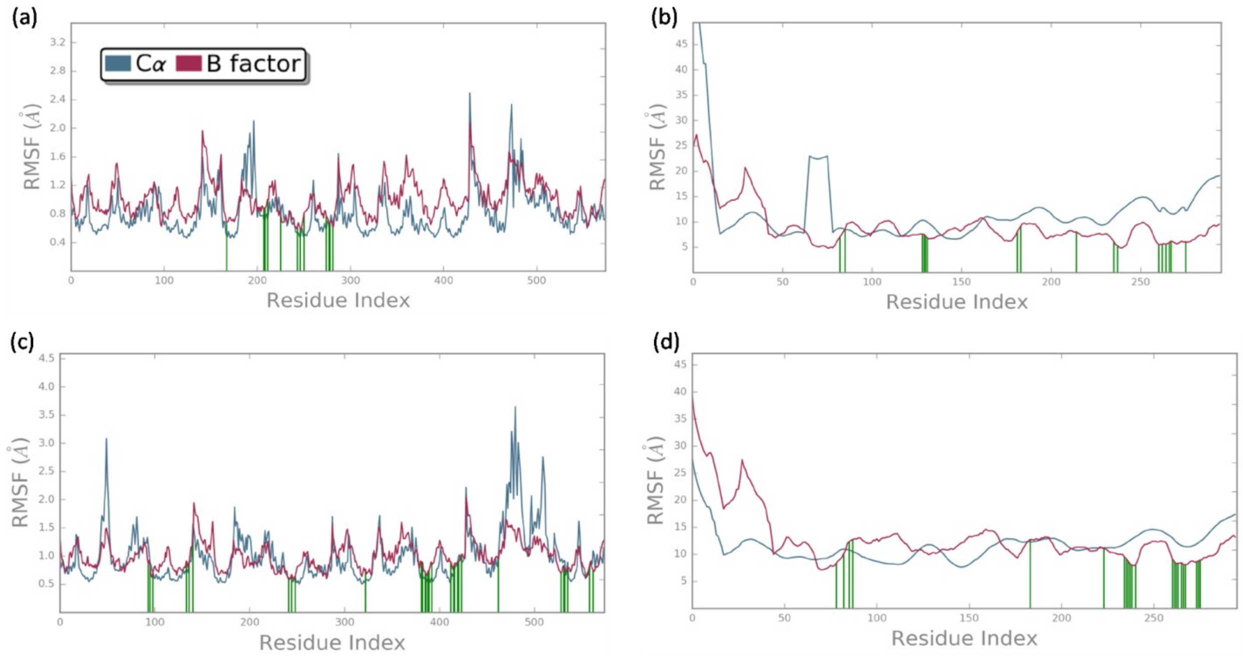

Figure. S5 Root mean square fluctuation plot with active site residual contacts of (a) hemoglobin complex with 1 decene (b) thrombin complex with 1 decene (c) hemoglobin complex with f127 (d) thrombin complex with f127.

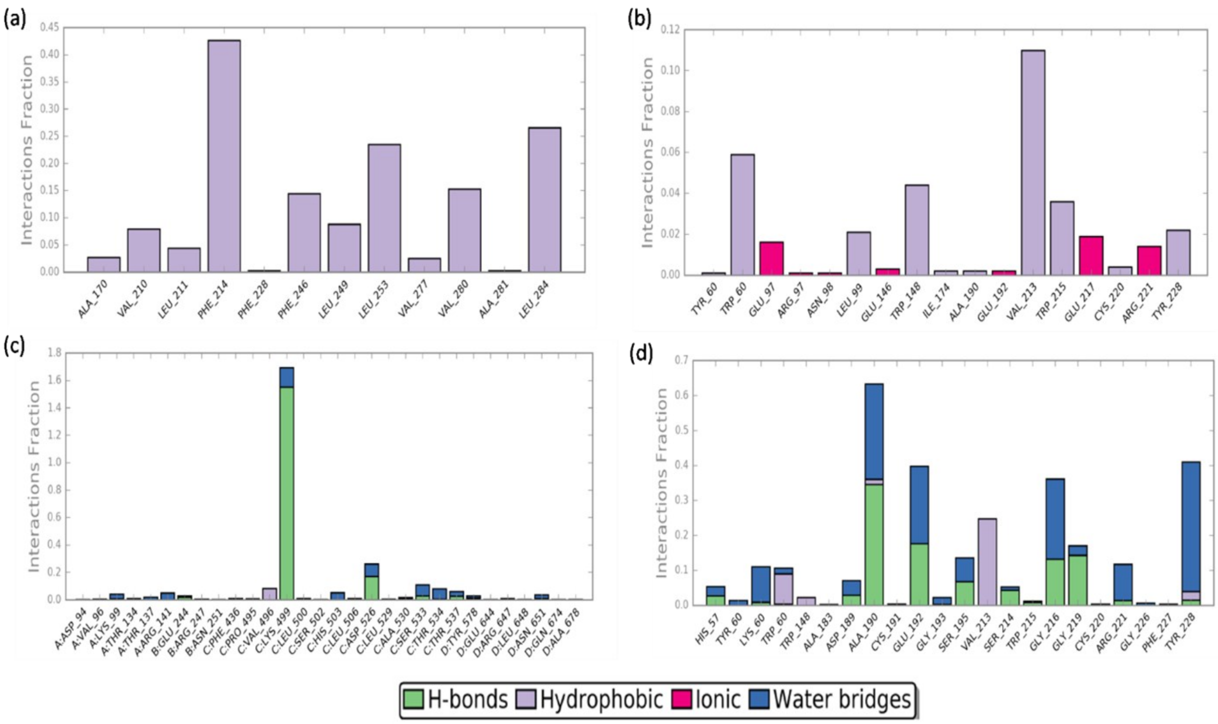

Figure. S6 Protein ligand contacts with active site residues during simulation (a) hemoglobin complex with 1 decene (b) thrombin complex with 1 decene (c) hemoglobin complex with f127 (d) thrombin complex with f127.

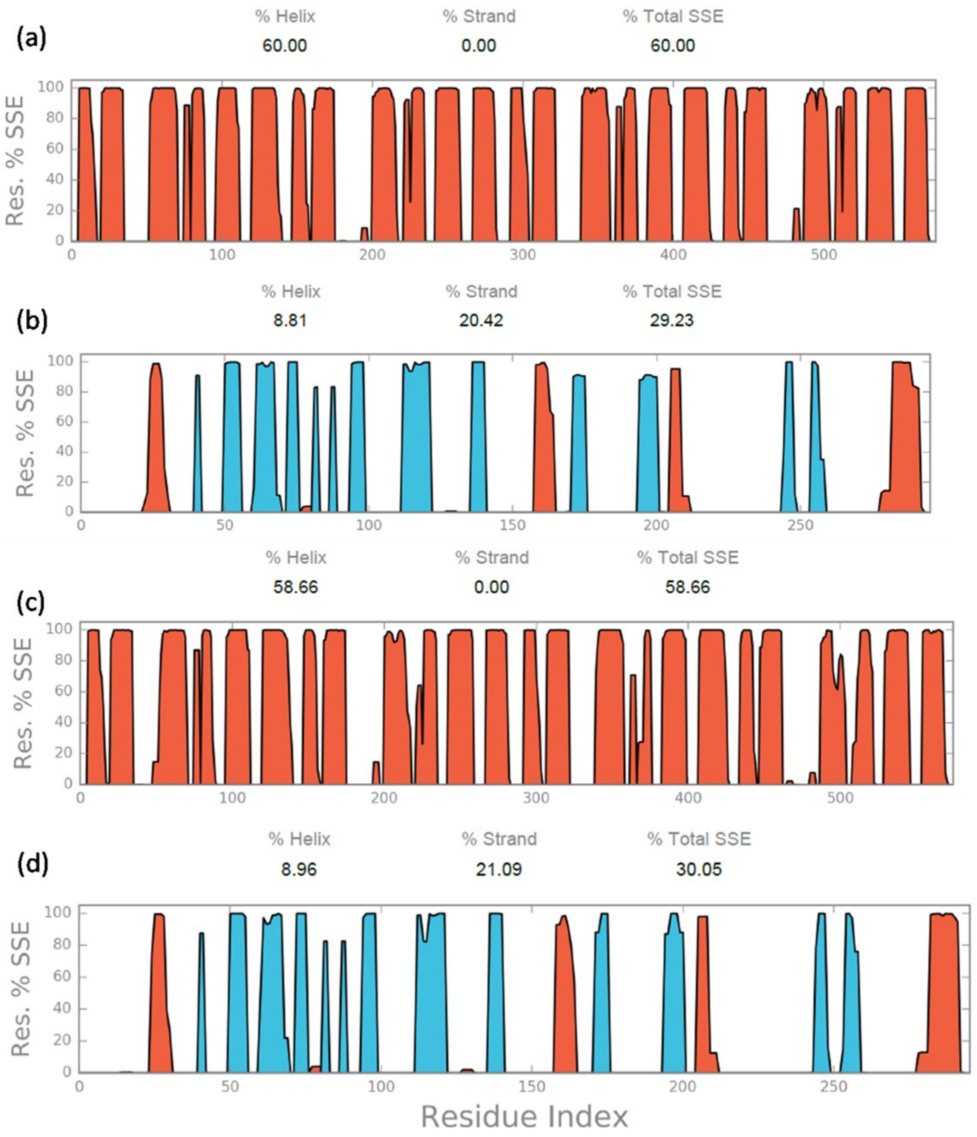

Figure. S7 Secondary structural simulations of (a) hemoglobin complex with 1 decene (b) thrombin complex with 1 decene (c) hemoglobin complex with f127 (d) thrombin complex with f127.

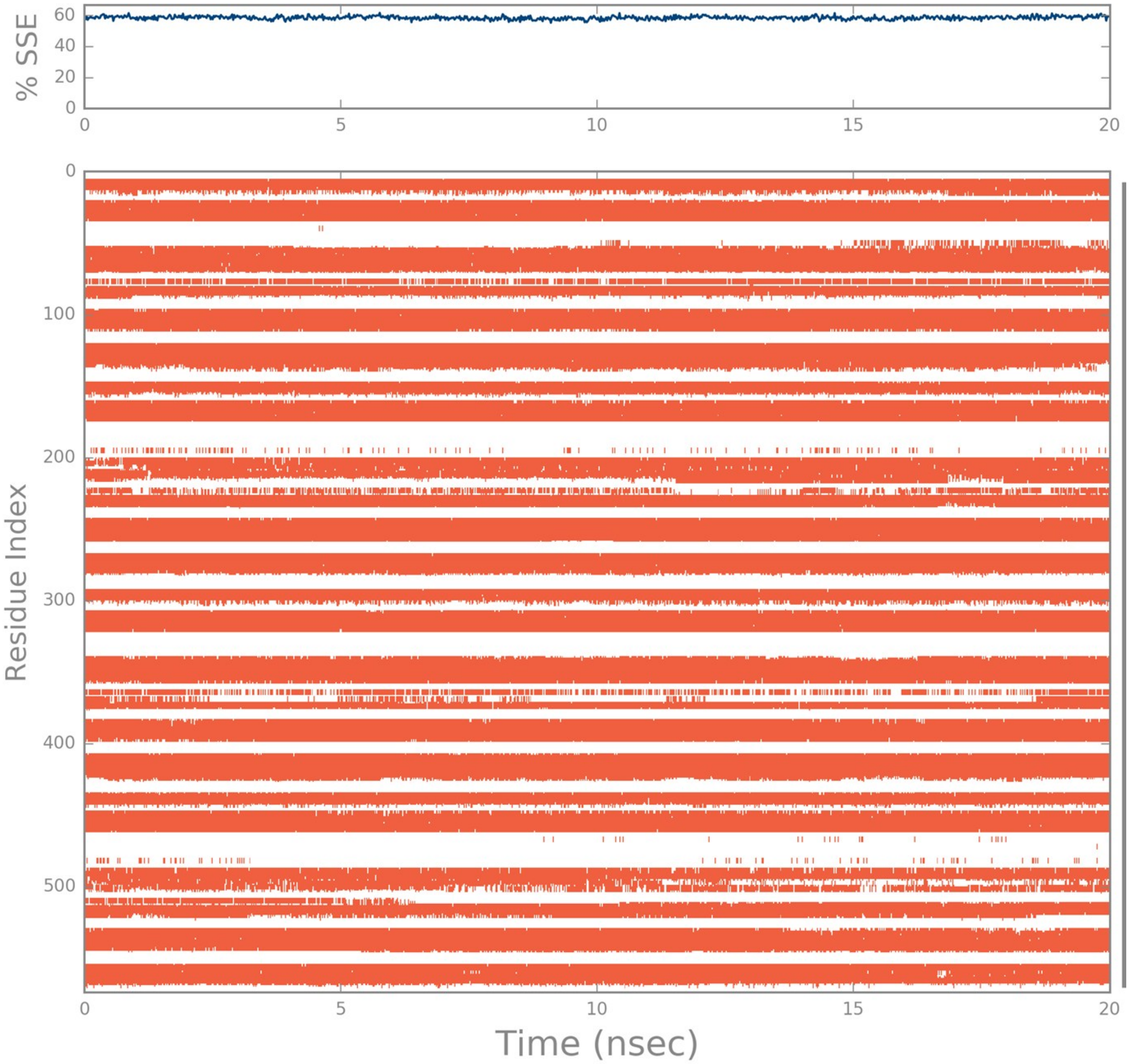

Figure. S8 The plot above summarizes the SSE (alpha-helices and beta-strands) composition for each trajectory frame over the course of the simulation, and the plot below monitors each residue and its SSE assignment over time for hemoglobin – f127 complex

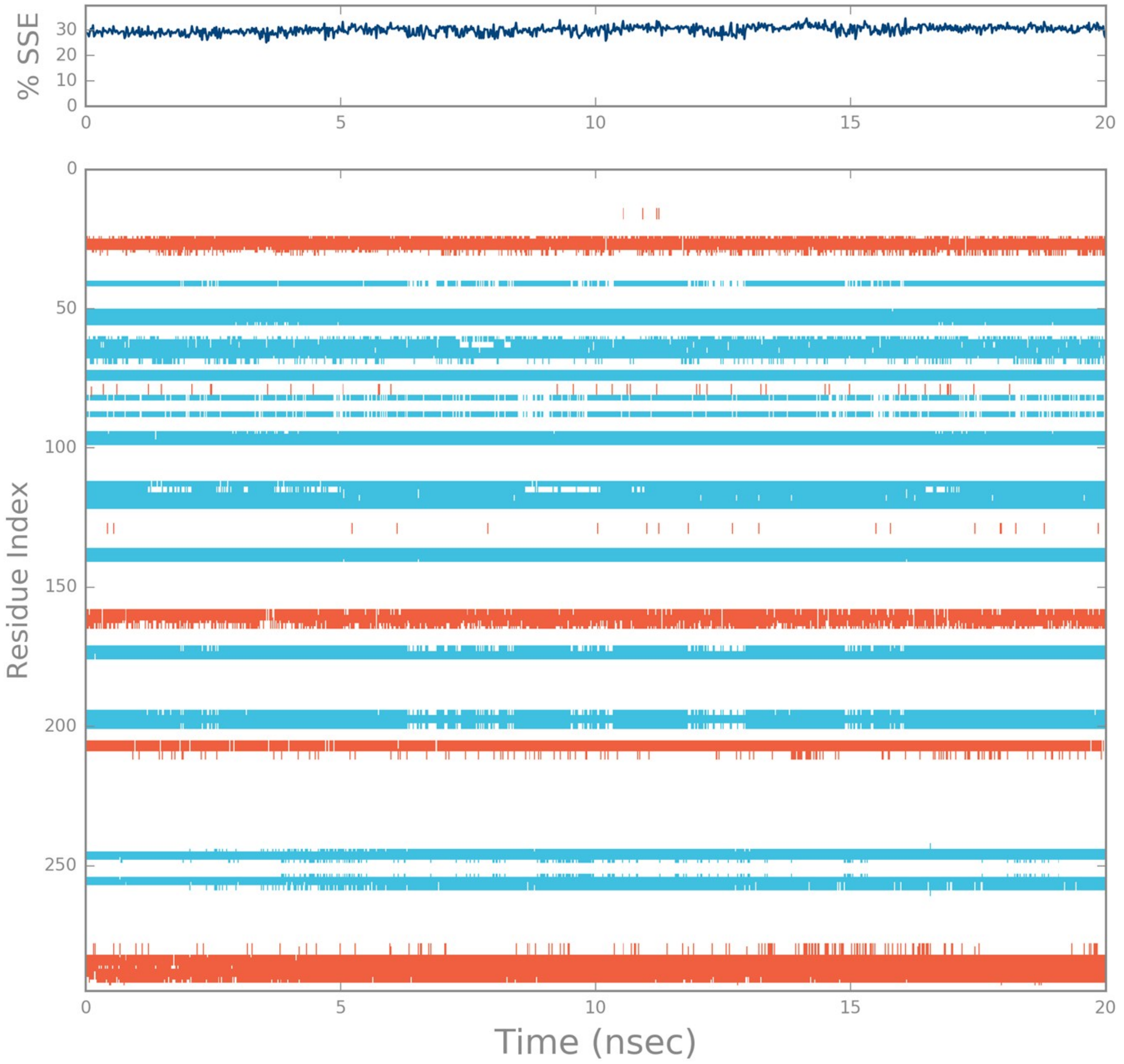

Figure. S9 The plot above summarizes the SSE (alpha-helices and beta-strands) composition for each trajectory frame over the course of the simulation, and the plot below monitors each residue and its SSE assignment over time for thrombin – f127 complex

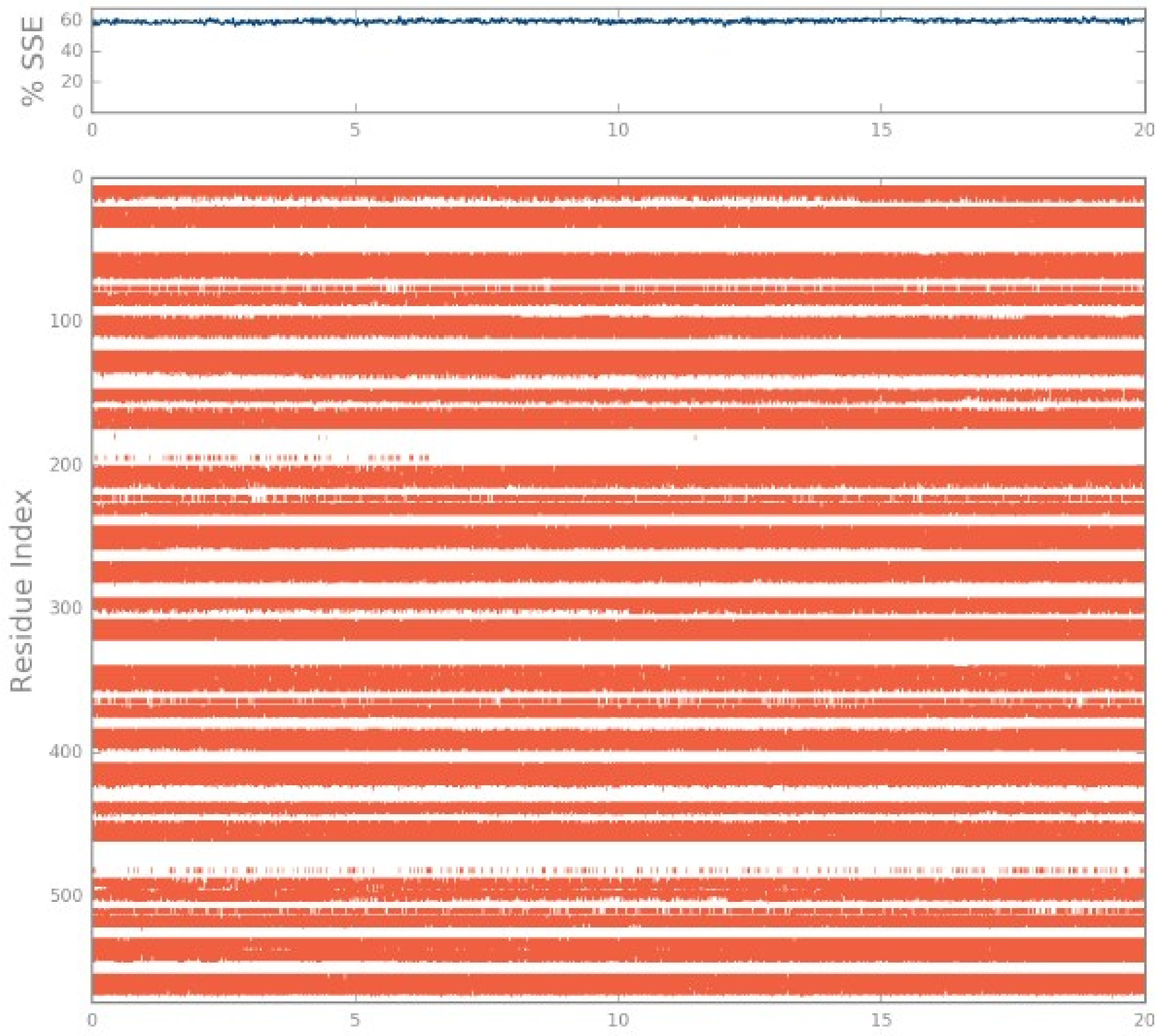

Figure. S10 The plot above summarizes the SSE (alpha-helices and beta-strands) composition for each trajectory frame over the course of the simulation, and the plot below monitors each residue and its SSE assignment over time for hemoglobin – 1decene complex

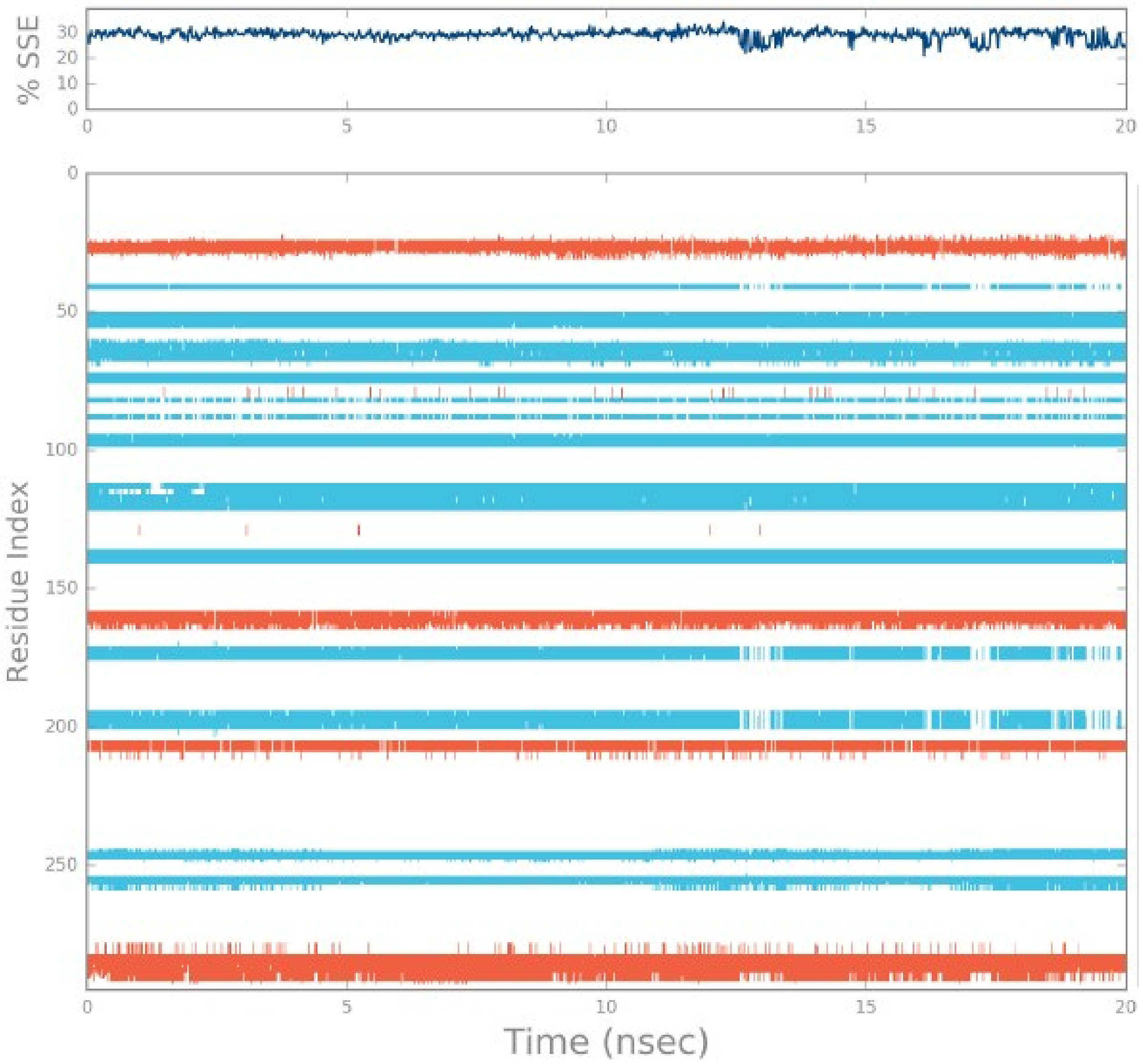

Figure. S11 The plot above summarizes the SSE (alpha-helices and beta-strands) composition for each trajectory frame over the course of the simulation, and the plot below monitors each residue and its SSE assignment over time for thrombin – 1decene complex

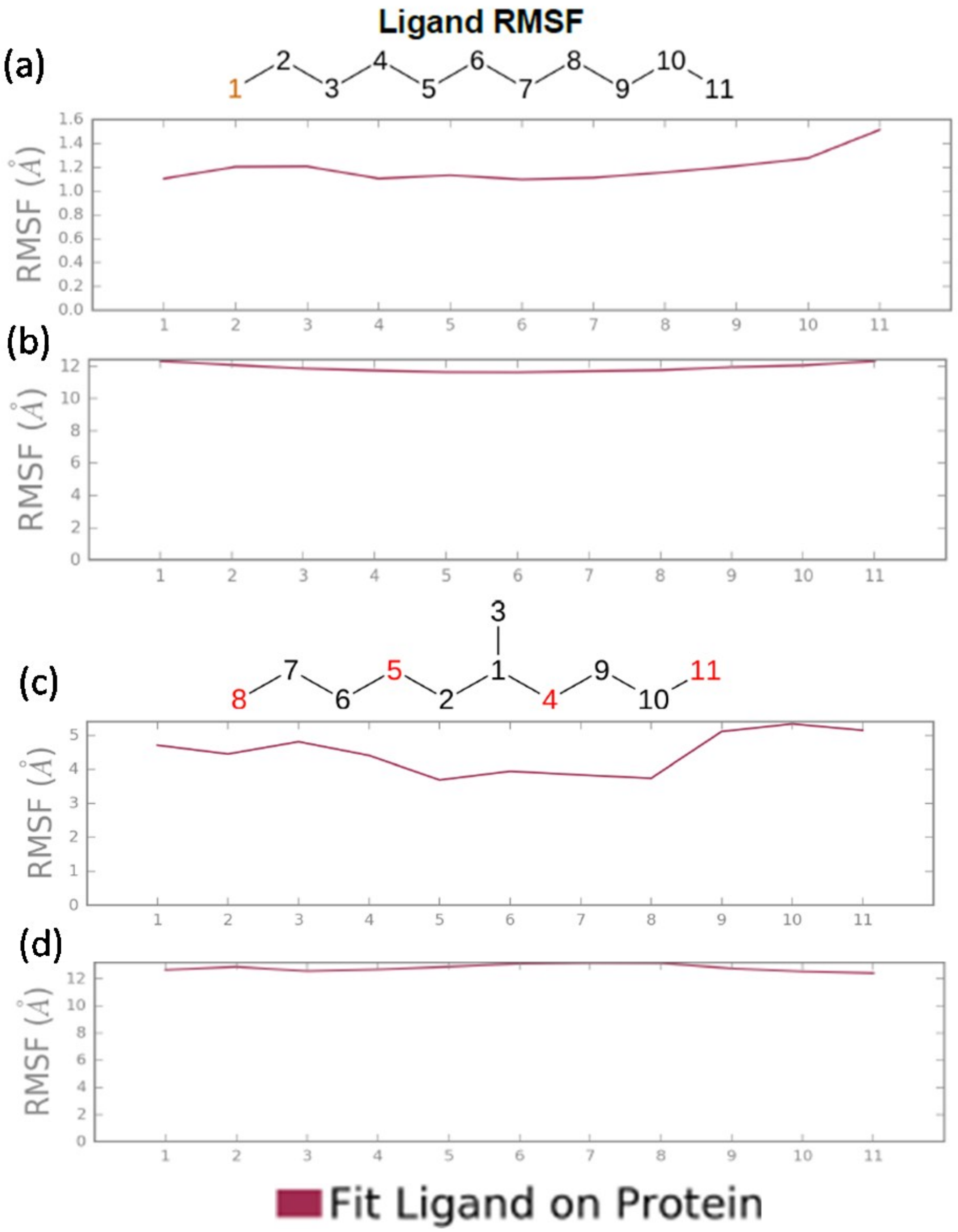

Figure. S12 Ligand fitting plot of (a) (a) hemoglobin complex with 1 decene (b) thrombin complex with 1 decene (c) hemoglobin complex with f127 (d)) thrombin complex with f127

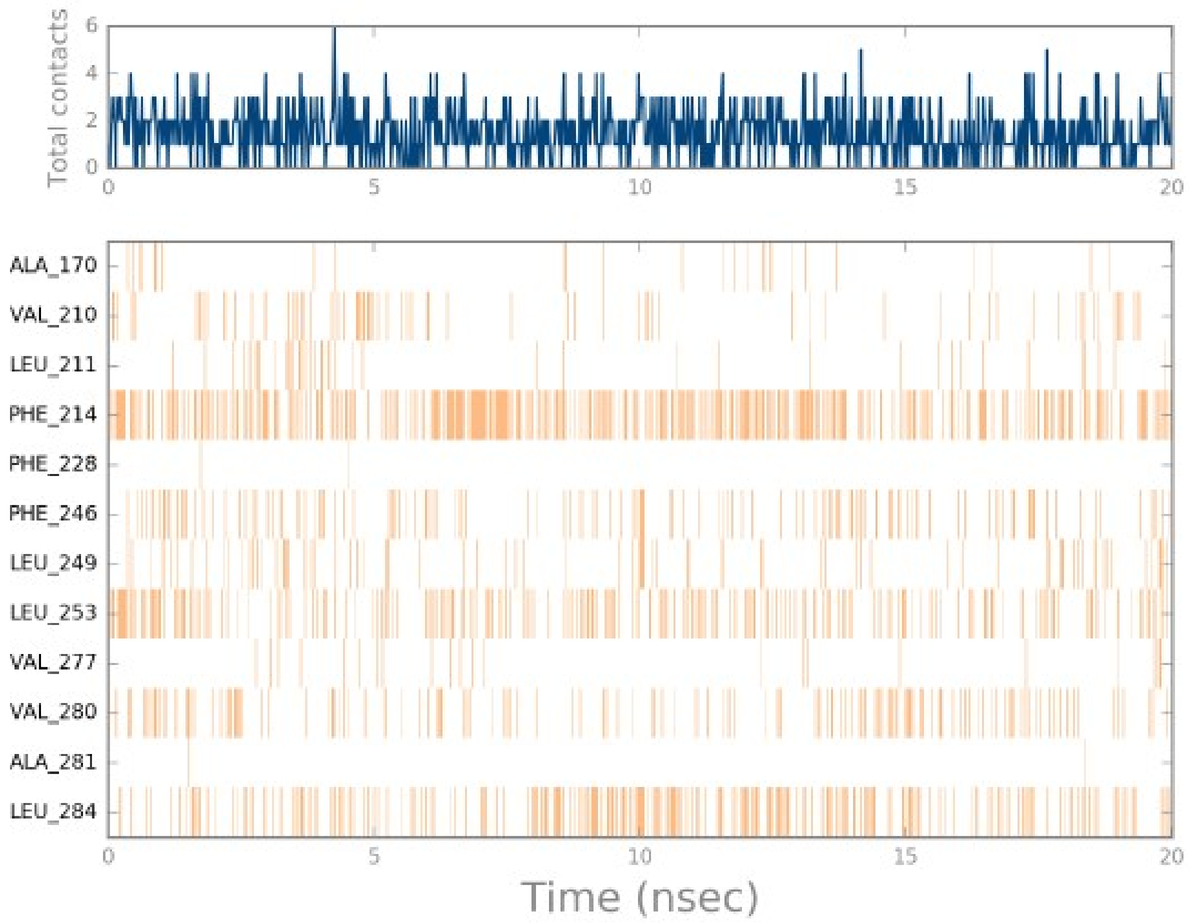

Figure. S13 A timeline representation of the interactions and contacts (**H-bonds**, **Hydrophobic**, **Ionic**, **Water bridges**) top panel shows the total number of specific contacts the protein makes with the ligand over the course of the trajectory. The bottom panel shows which residues interact with the ligand in each trajectory frame of hemoglobin – 1decene complex

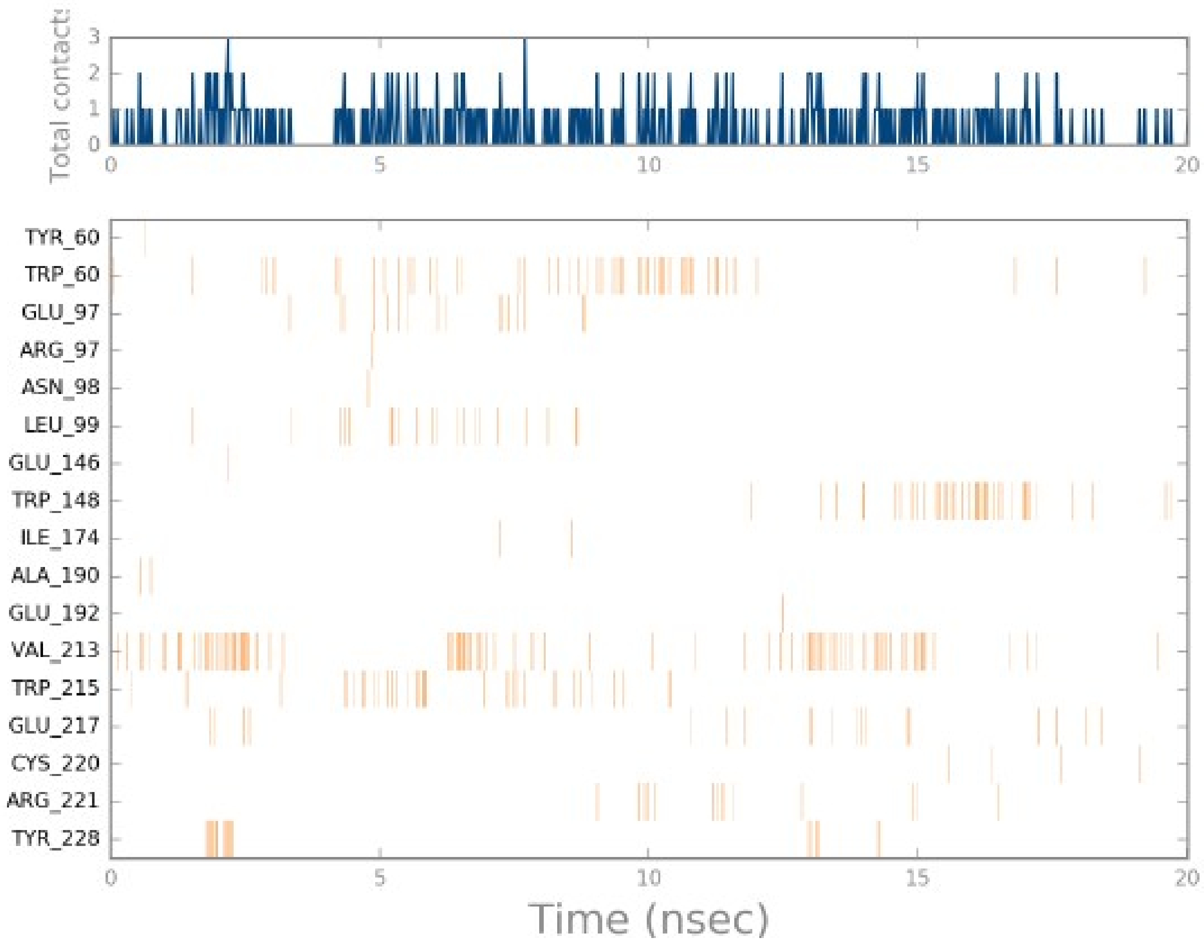

Figure. S14 A timeline representation of the interactions and contacts (**H-bonds**, **Hydrophobic**, **Ionic**, **Water bridges**) top panel shows the total number of specific contacts the protein makes with the ligand over the course of the trajectory. The bottom panel shows which residues interact with the ligand in each trajectory frame of thrombin – 1decene complex

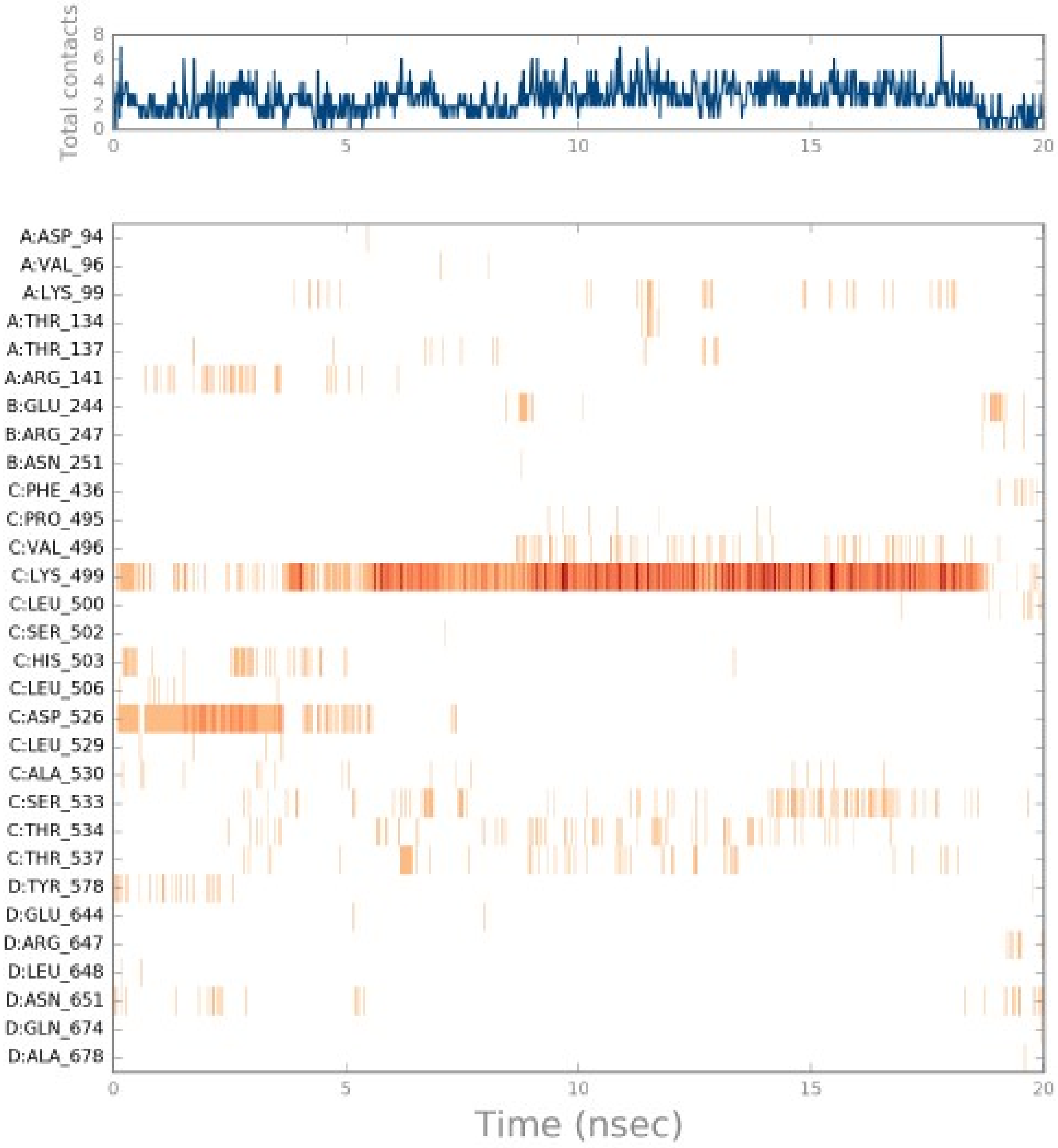

Figure. S15 A timeline representation of the interactions and contacts (**H-bonds**, **Hydrophobic**, **Ionic**, **Water bridges**) top panel shows the total number of specific contacts the protein makes with the ligand over the course of the trajectory. The bottom panel shows which residues interact with the ligand in each trajectory frame of hemoglobin – f127 complex

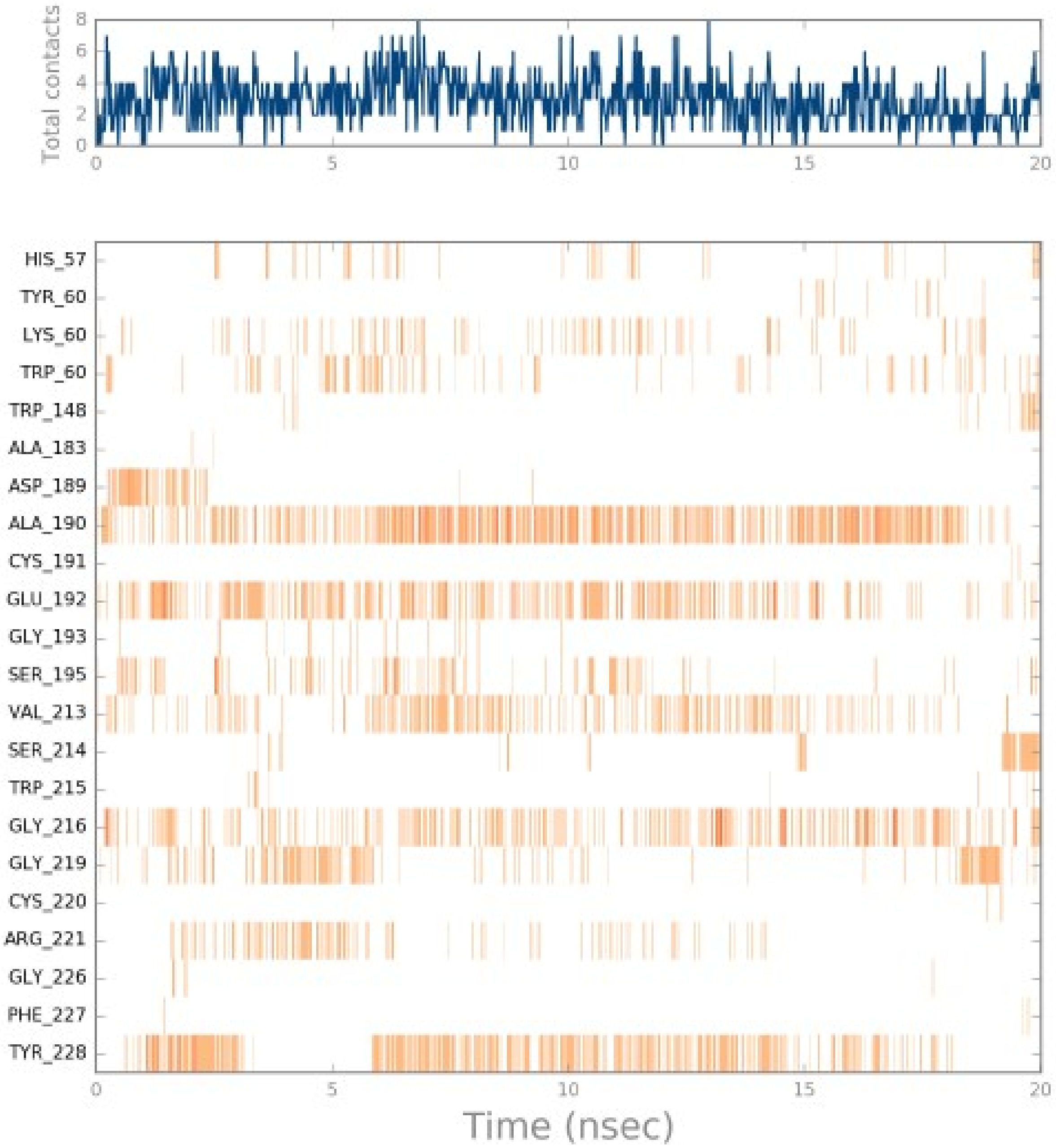

Figure. S16 A timeline representation of the interactions and contacts (**H-bonds**, **Hydrophobic**, **Ionic**, **Water bridges**) top panel shows the total number of specific contacts the protein makes with the ligand over the course of the trajectory. The bottom panel shows which residues interact with the ligand in each trajectory frame of thrombin – f127 complex

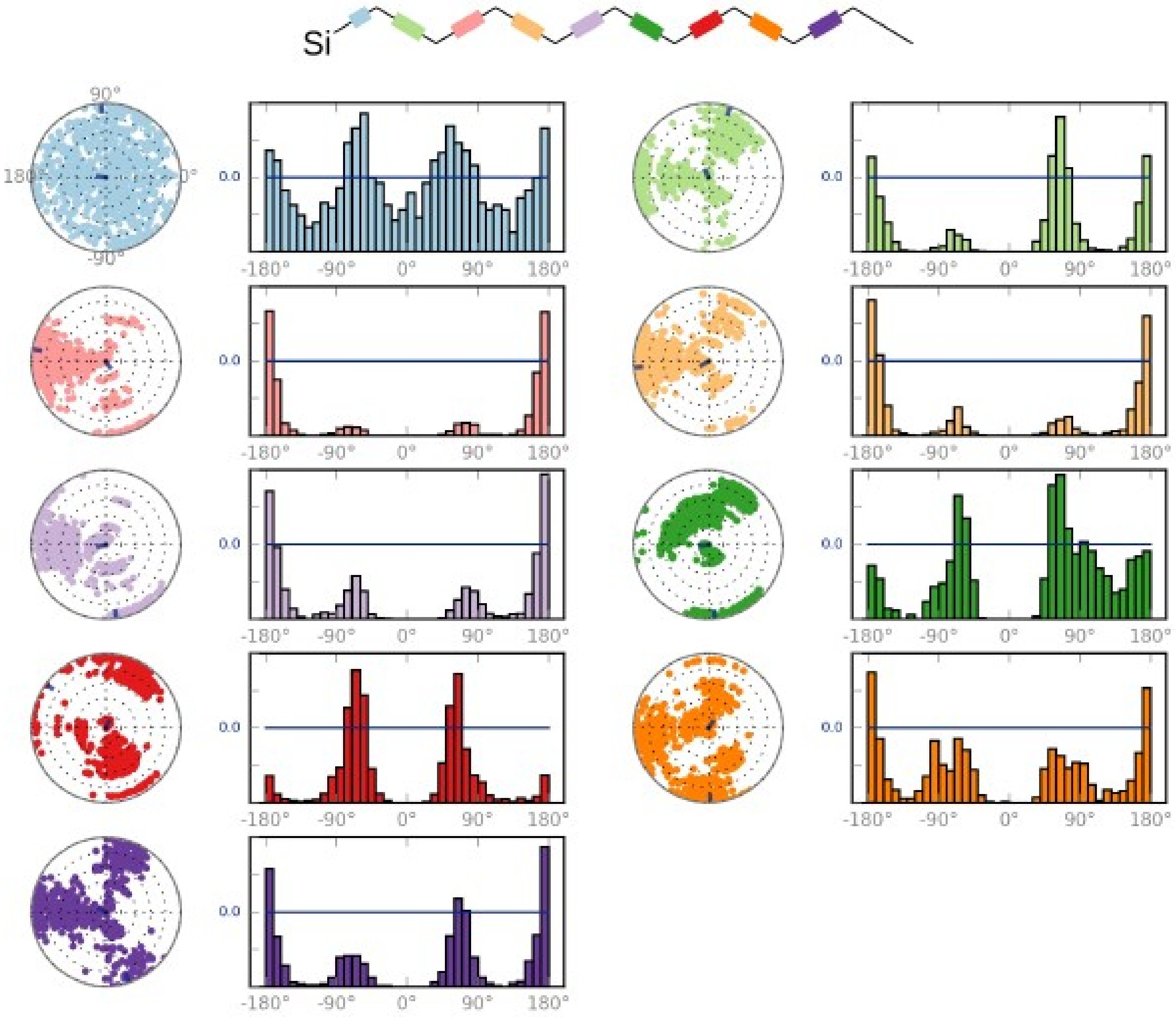

Figure. S17 The ligand torsions plot summarizes the conformational evolution of every rotatable bond (RB) in the ligand throughout the simulation trajectory for hemoglobin – 1decene complex (0.00 through 20.02 nsec). The top panel shows the 2d schematic of a ligand with color-coded rotatable bonds. Each rotatable bond torsion is accompanied by a dial plot and bar plots of the same color. Dial (or radial) plots describe the conformation of the torsion throughout the course of the simulation. The bar plots summarize the data on the dial plots, by showing the probability density of the torsion. The values of the potential are on the left *Y*-axis of the chart, and are expressed in *kcal/mol*. Looking at the histogram and torsion potential relationships may give insights into the conformational strain the ligand undergoes to maintain a hemoglobin – 1decene bound conformation

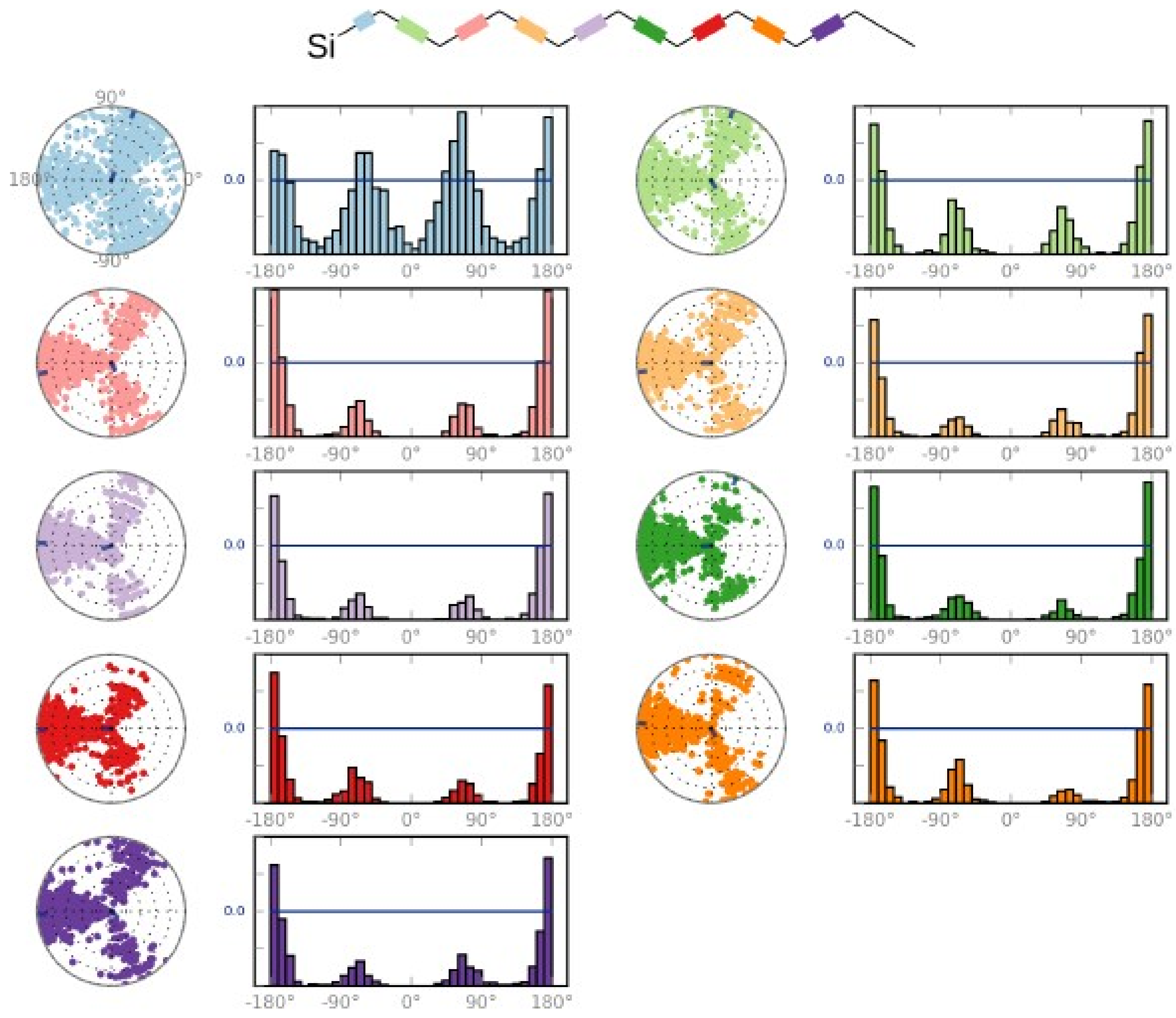

Figure. S18 The ligand torsions plot summarizes the conformational evolution of every rotatable bond (RB) in the ligand throughout the simulation trajectory for thrombin – 1decene complex (0.00 through 20.02 nsec). The top panel shows the 2d schematic of a ligand with color-coded rotatable bonds. Each rotatable bond torsion is accompanied by a dial plot and bar plots of the same color. Dial (or radial) plots describe the conformation of the torsion throughout the course of the simulation. The bar plots summarize the data on the dial plots, by showing the probability density of the torsion. The values of the potential are on the left *Y*-axis of the chart, and are expressed in *kcal/mol*. Looking at the histogram and torsion potential relationships may give insights into the conformational strain the ligand undergoes to maintain a thrombin – 1decene bound conformation

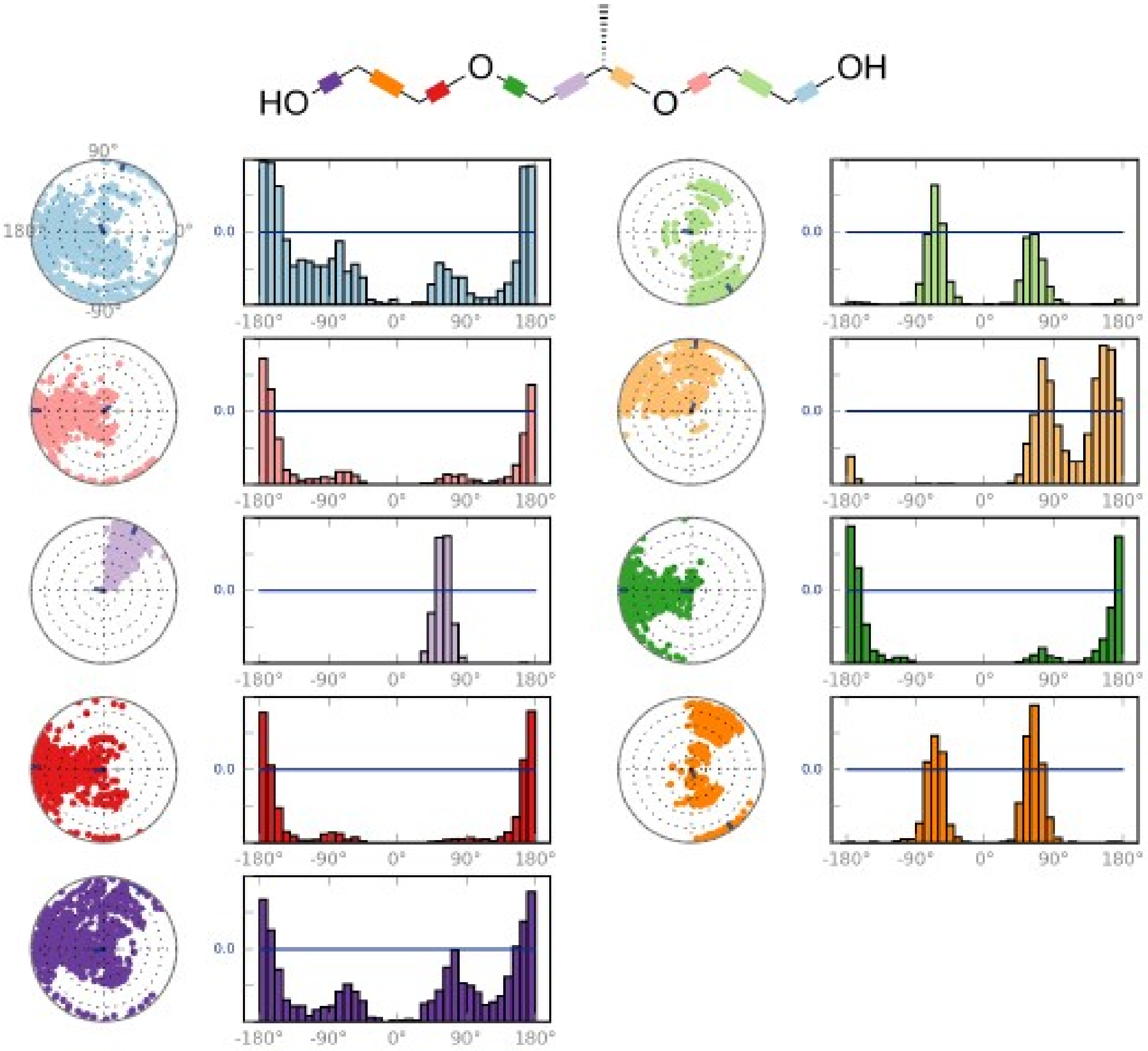

Figure. S19 The ligand torsions plot summarizes the conformational evolution of every rotatable bond (RB) in the ligand throughout the simulation trajectory for hemoglobin – f127 complex (0.00 through 20.02 nsec). The top panel shows the 2d schematic of a ligand with color-coded rotatable bonds. Each rotatable bond torsion is accompanied by a dial plot and bar plots of the same color. Dial (or radial) plots describe the conformation of the torsion throughout the course of the simulation. The bar plots summarize the data on the dial plots, by showing the probability density of the torsion. The values of the potential are on the left *Y*-axis of the chart, and are expressed in *kcal/mol*. Looking at the histogram and torsion potential relationships may give insights into the conformational strain the ligand undergoes to maintain a hemoglobin – f127 bound conformation

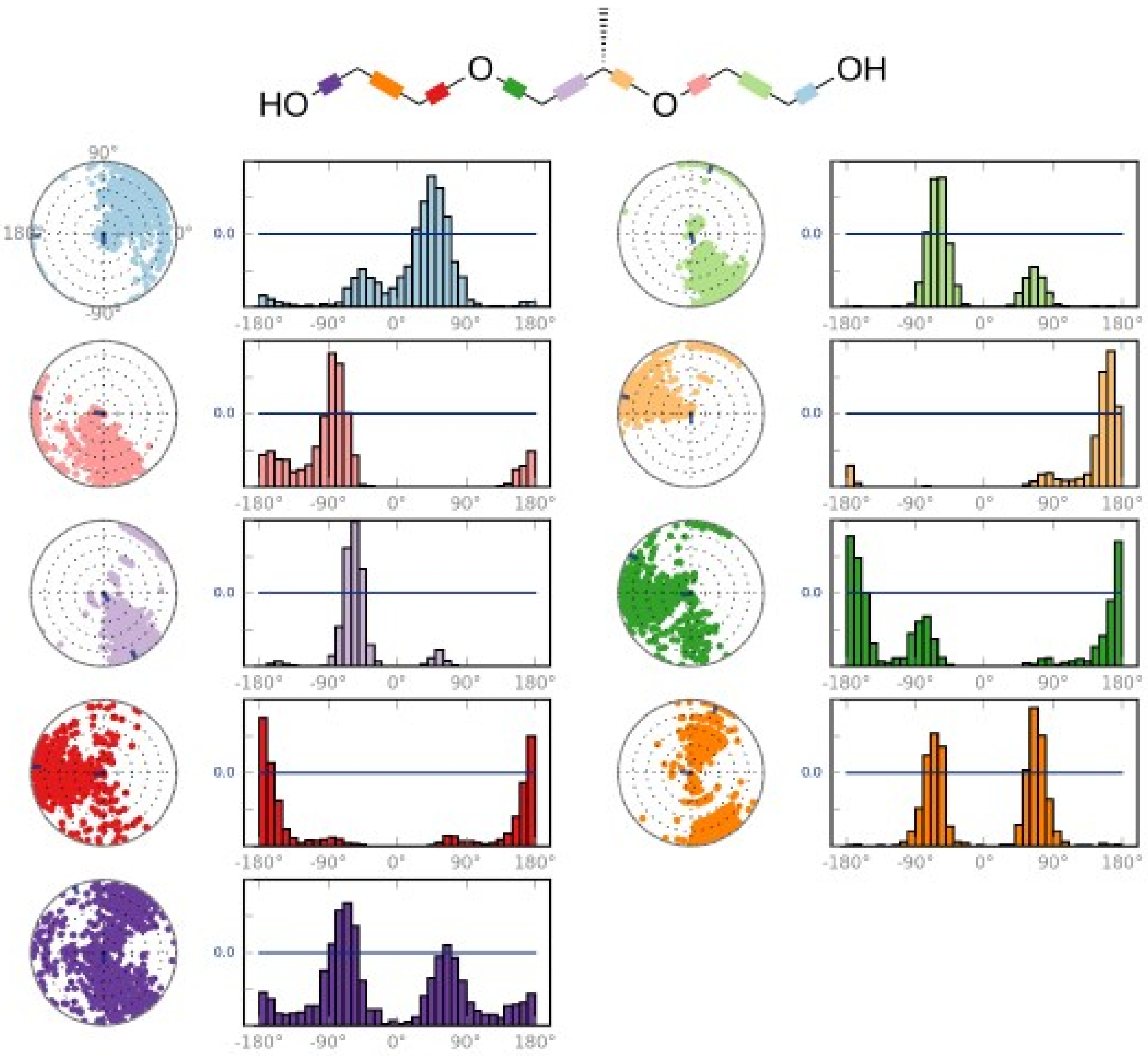

Figure. S20 The ligand torsions plot summarizes the conformational evolution of every rotatable bond (RB) in the ligand throughout the simulation trajectory for thrombin – f127 complex (0.00 through 20.02 nsec). The top panel shows the 2d schematic of a ligand with color-coded rotatable bonds. Each rotatable bond torsion is accompanied by a dial plot and bar plots of the same color. Dial (or radial) plots describe the conformation of the torsion throughout the course of the simulation. The bar plots summarize the data on the dial plots, by showing the probability density of the torsion. The values of the potential are on the left *Y*-axis of the chart, and are expressed in *kcal/mol*. Looking at the histogram and torsion potential relationships may give insights into the conformational strain the ligand undergoes to maintain a thrombin – f127 bound conformation
